# A Comprehensive Analysis of the Effect of Histological Subtypes on the Survival Probability of Kidney Carcinoma Patients: A Hypertabastic Survival Analysis

**DOI:** 10.36959/896/604

**Published:** 2020-12-28

**Authors:** Mohammad Tabatabai, Stephanie Bailey, Patricia Matthews-Juarez, Habib Tabatabai, Nader Bahri, Lyle Cooper, Derek Wilus, Karan Singh, Paul Juarez

**Affiliations:** 1 Meharry Medical College, Nashville, USA; 2 University of Wisconsin Milwaukee, Milwaukee, USA; 3 University of Texas Health Sciences Center, Tyler, USA

**Keywords:** Histological subtypes, Hypertabastic survival models, kidney carcinoma, tumor grade, tumor stage

## Abstract

The purpose of this study is two-fold. First, to find out whether the histological subtypes can serve as an independent prognostic factor for kidney carcinoma; and second, whether it’s role can be maintained when we control for confounders. Using National Cancer Institute data from 1975-2016, we have modeled the impact of histological subtypes on the survival probability of kidney carcinoma patients. A total of 134,150 individuals were examined from the Surveillance, Epidemiology, and End Results program (SEER) [1]. The study variables are age, race/ethnicity, sex, tumor grade, type of surgery, geographical location of patient and stage of disease. We have applied the Hypertabastic proportional hazards survival model [2-6] to analyze the survival time of patients diagnosed with kidney carcinoma in order to explore the effect of histological subtypes on their survival probability. In particular, our intention was to assess the relationship between the histological subtypes and tumor stage, grade, and type of surgery. Our results indicated that histology plays an important role both when used as the sole predictor in the survival model (*P* < 0.001), as well as when controlling for confounding variables (*P* < 0.001).

## Background

Kidney carcinoma is caused when healthy cells in one or both kidneys begin to grow rapidly form a tumor. According to the American Cancer Society’s prediction of new cases of kidney cancer, which include renal pelvis cancer in the United States for the year 2020, 14,830 of 73,750 patients are predicted to die [7]. Risk factors for developing kidney carcinoma includes, but is not limited to, hypertension, smoking, obesity, hepatitis C and presence of other kidney diseases [8]. There has been an increasing trend in the rate of new kidney cancers since 1990 [7]. In 2016, the incidence rate of kidney and renal pelvis cancers was 16.8 per 100,000 people, which was ranked 9^th^ among cancer incidence rates [9].

Histological differences play a significant role in prediction as well as diagnoses of kidney carcinoma and have a major impact in the survival time of such patients [10]. There are several types of kidney carcinoma including renal cell carcinoma (RCC), urothelial carcinoma, and sarcoma. RCC is the most common type, making up about 85% of diagnoses. Urothelial carcinoma, sometimes called transitional cell carcinoma, accounts for about 5-10% of diagnoses; whereas sarcoma is a rare type of kidney cancer accounting for less than 5% of all cases [11]. Within RCC, there are different histological subtypes, which are identified by a pathologist. Chromophobe RCC accounts for approximately five percent of RCC tumors [12]. This histological subtype is normally less aggressive when compared with the other types of RCCs. Chromophobe RCC tumors can become large, but tend to stay localized. There is no significant difference in the incidence rates of chromophobe RCC tumors between males and females, with the survival probability of this cancer being quite high [12,13]. Papillary RCC accounts for 10-15% of patients diagnosed with RCC [14]. Clear cell is the most common form of RCC.

Individuals who have been diagnosed with papillary RCC have lower risk of death, when compared to clear cell RCC [15]. Sarcomatoid RCC tends to grow more quickly than other types of kidney cancer. This rapid growth, makes the treatment more difficult and increases the likelihood of becoming a metastatic cancer [16]. Kang, et al. analyzed the conditional survival probability of patients with distant RCC who were treated with tyrosine kinase inhibitor therapy and identified predictors of conditional survival [17]. Prognostic influence of histological subtypes and grading on the survival probability of RCC patients have long been studied in literature [18-20]. Genomic and survival data analyses were used to find the association between sirtuin family members and clear cell RCC [21]. Nguyen, et al. investigated the effect of histological subtypes on the survival of patients diagnosed with RCC, and found histological subtypes to be an important prognostic factor [22,23]. Hematology and histology play a vital role as predictive measures of RCC [24]. Teloken, et al. evaluated the effect of histological subtypes on patients diagnosed with localized RCC and concluded that clear cell RCC was a prognostic factor for patients who underwent surgery [25]. Kappa statistics were used to investigate the concordance between tumor histological subtypes at their original point of diagnosis and after slide revision [26]. A study by Cai, et al. investigated the effect of age on the survival probability of patients with localized RCC who underwent radical nephrectomy and found older age is associated with lower survival probability [27]. Carrasco, et al. studied the impact of histology on the survival of patients diagnosed with distant RCC who underwent cytoreductive nephrectomy [28], and Wagner, et al. compared the risk of death of clear cell RCC patients with those diagnosed with papillary RCC [15]. Both Carrasco and Wagner found the significance of histological subtypes in the treatment of RCC patients. In our study, we have analyzed the impact of histological subtypes on the survival probability of patients diagnosed with kidney carcinoma from 1975 to 2016 using the Hypertabastic proportional hazards model [2-6]. This parametric method is flexible and can accommodate different hazard shapes, and enable the researcher to better understand the effect of histological subtypes on the survival time of kidney carcinoma patients. Study results will be used to train medical students in understanding the extent of histological subtypes as a prognostic factor for patients diagnosed with kidney carcinoma.

## Methods

The clinical, socio-economic, and histological study variables were age, race/ethnicity, sex, tumor grade, type of surgery, geographical location of patient, and stage of disease. This retrospective study examined a total of 134,150 individuals from the Surveillance, Epidemiology, and End Results program (SEER) data base of whom 85,324 were males and 48,826 were females. We applied the univariable and multivariable Hypertabastic proportional hazards survival model to analyze the survival time of patients diagnosed with kidney carcinoma. In addition, the complex relationship between histological subtypes and the study variables were examined. The histological subtypes were classified using methods provided by the International Classification of Diseases on Oncology, Third Edition (ICD-O-3). The following software were used: SAS 9.4, SPSS 26, ggplot2 in R Studio 1.3.1073, and Mathematica 12.

## Results

A review of our data indicated that the overall mean (SD) and median age for our cohort was 61.51 (12.59) and 62 years respectively. The mean (SD) and median ages were 61.29 (12.23) and 62 years for males, and 61.89 (13.19) and 63 years for females, respectively. There was a significant difference in mean age between male and females (P-value < 0.001). Data revealed 57.8% of partients had adeno carcinoma with mixed subtype, 19.7% had papillary adeno carcinoma NS, 11.1% suffered from clear cell adeno carcinoma, 3.9% had renal cell adeno carcinoma, 2.5% were identified as having chromophobe RCC, 1.1% had sarcomatoid RCC, and only 0.7% were diagnosed with granular cell carcinoma. These seven histological subtypes included approximately 97% of the individuals in our study. The remaining 59 histological subtypes accounted for only 3% of the study’s patients and their group is named “other histological types.” Table 1 provides the name of all histological subtypes considered in this study together with their corresponding frequency and median survival time.

**Figure 1:**
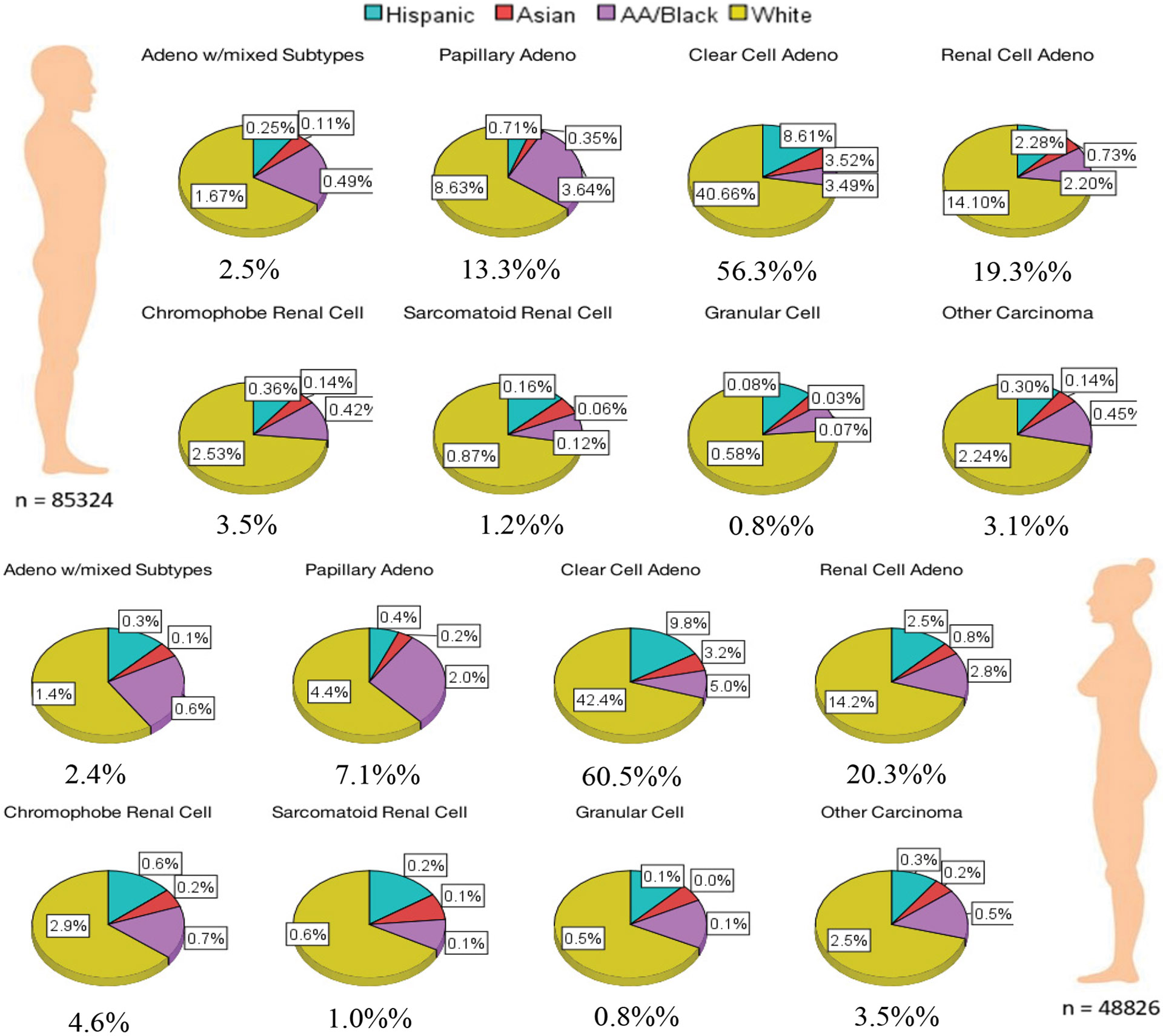
Racial distribution based on histological subtypes and sex.

For all histological subtypes considered in our study, the percentage of males was higher than females (almost twice as high in males as in females in most cases). For instance, among individuals diagnosed with clear cell adenocarcinoma, 38.1% were female and 61.9% were male. For both males and females, clear cell adenocarcinoma had the highest percentage among the histological subtypes (60.5% female and 56.3% male). Among males and females with kidney carcinoma, 40.66% and 42.4% had clear cell adenocarcinoma and were White respectively. Among males and females with kidney carcinoma, 8.61% and 9.8% had clear cell adeno carcinoma and were Hispanic respectively. Among males and females with kidney carcinoma, 3.49% and 5.0% had clear cell adenocarcinoma and were AA/Black respectively. Among males and females with kidney carcinoma, 3.52% and 3.2% had clear cell adenocarcinoma and were Asian respectively. Figure 1 illustrates the racial distribution of eight histological subtypes for males and females.

The stage of tumor helps in understanding the seriousness of kidney carcinoma. It improves estimation of prognosis and will eventually assist health care providers in their plan of treatment in order to increase the survival of their patients. Our data revealed that 74% of patients had localized, 17.3% had regional, and 8.7% had distant tumor stage. As indicated in Table 2, within all tumor stages, the majority of patients were diagnosed with clear cell adenocarcinoma. Within localized tumor stage, the lowest percentage of patients were diagnosed with sarcomatoid RCC; and within regional tumor stage, the lowest percentage belonged to granular cell carcinoma. Chromophobe RCC and granular cell carcinoma had the lowest percentages of patients within distant tumor stage. With the exception of sarcomatoid RCC, the number of patients diagnosed with localized tumor stage was the highest among all histological subtypes. Among sarcomatoid RCC patients, the percentage diagnosed with distant tumor stage was the highest.

The racial composition of our patients’ data is comprised of 13.3% Hispanic, 5% Asian/Pacific Islanders, 11.2% African American, and 70.5% White. Among African Americans, the percentage of patients diagnosed with clear cell adeno carcinoma was highest, followed by papillary adenocarcinoma NOS, and renal cell adenocarcinoma; however, granular cell carcinoma had the lowest percentage, as seen in Table 3. Among Whites, Asian/Pacific Islanders, and Hispanics, the percentage of patients diagnosed with clear cell adeno carcinoma was highest, followed by renal cell adenocarcinoma. The lowest percentage among these three racial groups was granular cell carcinoma. Although the percentage of patients diagnosed with clear cell adenocarcinoma for all racial groups was highest when compared with all histological subtypes, the percentages of Whites, Asian/Pacific Islanders, and Hispanics were approximately 1.6, 1.9, and 1.9 times higher than that of African Americans, respectively. Among all racial groups, African Americans had the lowest percentage of clear cell adeno carcinoma. Although African Americans only accounted for 11.2 percent of our data, they were vastly overrepresented in those diagnosed with papillary adenocarcinoma as well as those with adenocarcinoma with mixed subtypes.

The regional distribution of patients residing in the Pacific Coast, East, Northern Plains, and Southwest was 45.5%, 39.4%, 10.8%, and 4.4% respectively. The percentage of patients diagnosed with clear cell adeno carcinoma across all regions were at least 50%; whereby the lowest percentage across all regions belonged to patients who were diagnosed with granular cell carcinoma. As shown in Table 4, the majority of patients diagnosed with renal cell adeno carcinoma were from the East region. Within granular cell carcinoma patients, 27.3% were from the East region, whereas 57% were from the Pacific Coast region. East had the highest percentage of renal cell adenocarcinoma, papillary adeno carcinoma NOS, and other histological subtypes; whereas Pacific Coast had the highest percentage for the remaining subtypes.

The distribution of tumor grade was as follows: 50.6% moderately differentiated, 28.2% poorly differentiated, 13.5% well differentiated, and 7.6% undifferentiated. Among all tumor grades, clear cell adeno carcinoma had the highest percentage of patients, followed by renal cell adeno carcinoma. For all tumor grades except undifferentiated, papillary adenocarcinoma had the third highest percentage of patients. For undifferentiated tumor grade, the highest percentage belonged to clear cell adenocarcinoma followed by renal cell carcinoma and sarcomatoid RCC, respectively. For all histological subtypes, with the exception of sarcomatoid RCC and the Other histological types group, the percentage of patients who had moderately differentiated tumor grade was highest. For sarcomatoid RCC, the highest percentage belonged to undifferentiated grade; and for Other histological types group, poorly differentiated grade had the highest percentage. For adeno carcinoma patients with mixed subtypes, sarcomatoid RCC, granular cell carcinoma, and the Other histological types group, the well differentiated tumor grade had the lowest percentage. With regard to papillary adenocarcinoma, clear cell adeno carcinoma, renal cell adenocarcinoma, and chromophobe RCC, the undifferentiated tumor grade had the lowest percentage, as indicated in Table 5.

The distribution of surgery types were as follows: 4% had no surgery, 1% had cryosurgery 13 (local tumor destruction, NOS), 0.6% had thermal ablation, 1% had cryosurgery 23 (any combination of local tumor excision, polypectomy, or excisional therapy, NOS), 27.5% had partial nephrectomy or partial ureterectomy, 7.9% had complete nephrectomy, 55.7% had radical nephrectomy, 1% had any nephrectomy, 0.9% had nephrectomy, ureterectomy, and 0.7% had other types of surgery. With regard to all types of surgery, clear cell adenocarcinoma had the highest percentage. Within each histological subtype, radical nephrectomy had the highest percentage across all types of surgery, as shown in Table 6.

**Figure 2:**
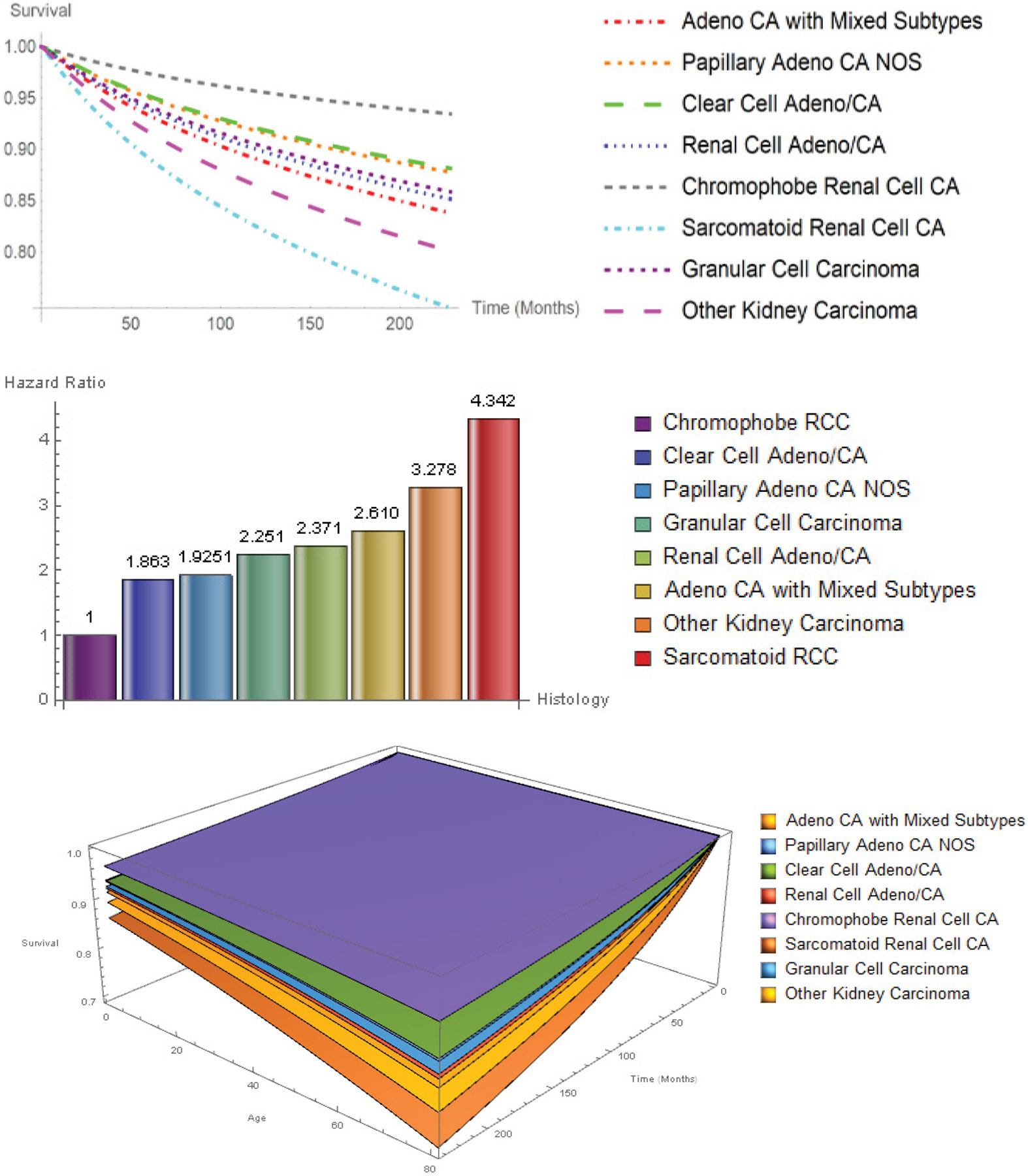
2D and 3D survival curves and hazard ratios for histological subtypes.

In the univariate Hypertabastic survival analyses, all factors were found to be independently significant (all *P* < 0.001). However, in the multivariable model, age, race, histology, stage, region, grade, and surgery type remained statistically significant (*P* < 0.001), with the exception of sex (*P* = 0.306). African Americans had significantly lower survival probabilities followed by Hispanics, Asian/Pacific Islanders and Whites. There was no statistically significant difference between survival probability of Asian/Pacific Islanders and Whites (*P* = 0.208). African Americans had the highest percentage among all racial groups in the following histological types: Papillary adenocarcinoma (27.2%), renal cell adenocarcinoma (21.4%), chromophobe RCC (4.7%), adenocarcinoma with mixed subtypes (4.6%), and other carcinomas (4.2%). In addition, African Americans had the highest percentage of no surgery (4.7%), complete nephrectomy (8.8%), nephrectomy/ureterectomy (1%), poorly differentiated (30.1%) cancer type, and localized (81.1%) tumor stage within race. Asian/Pacific Islanders had the highest distant (10.2%) tumor stage, while Hispanics had the highest regional (18.4%) tumor stage. The 5-year overall percentage of patients who survived was 93.8%. Figure 2 shows the survival probability curves as a function of survival time and the 3D survival probabilities as a function of survival time and age for eight histological subtypes. Sarcomatoid RCC (HR: 4.342, CI: 3.982-4.735) had the worst prognosis, followed by other (HR: 3.278, CI: 2.863-3.754), adeno with mixed subtypes (HR: 2.61 CI: 2.374-2.869), renal cell carcinoma (HR: 2.371, CI: 2.227-2.524), granular cell (HR: 2.251, CI: 1.256-2.590), papillary (HR: 1.925, CI: 1.772-2.091), clear cell adeno (HR: 1.863, CI: 1.753-1.980). The chromophobe RCC patients had the best probability of survival. All hazard ratios were calculated with respect to chromophobe RCC, as indicated in Figure 2.

**Figure 3:**
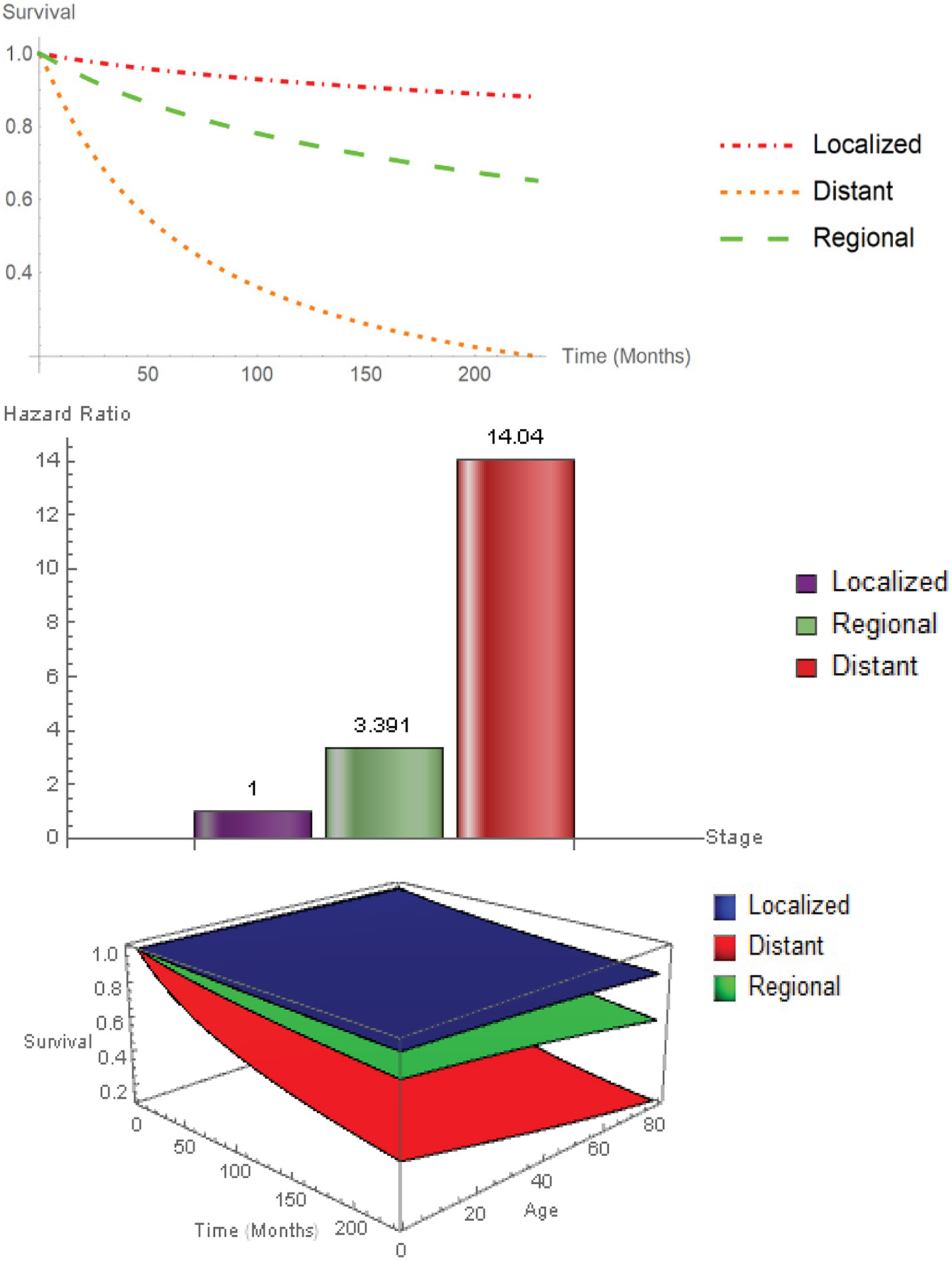
2D and 3D survival curves and hazard ratios for Stage.

Similarly, Figure 3 illustrates the survival probabilities and hazard ratios for tumor stage. Distant stage had the worst probability of survival, followed by regional. The best survival probability was for the localized tumor stage. The slope of the survival curve for patients diagnosed with distant stage is much higher when compared with regional stage and localized stage. This is reflected in the distant (HR: 14.04, CI: 13.515-14.585) and regional (HR: 3.391, CI: 3.261-3.523) stage hazard ratios, using localized stage as a reference. Patients with distant tumor stage had a 14.04-fold higher rate of death when compared to patients with localized tumor stage.

**Figure 4:**
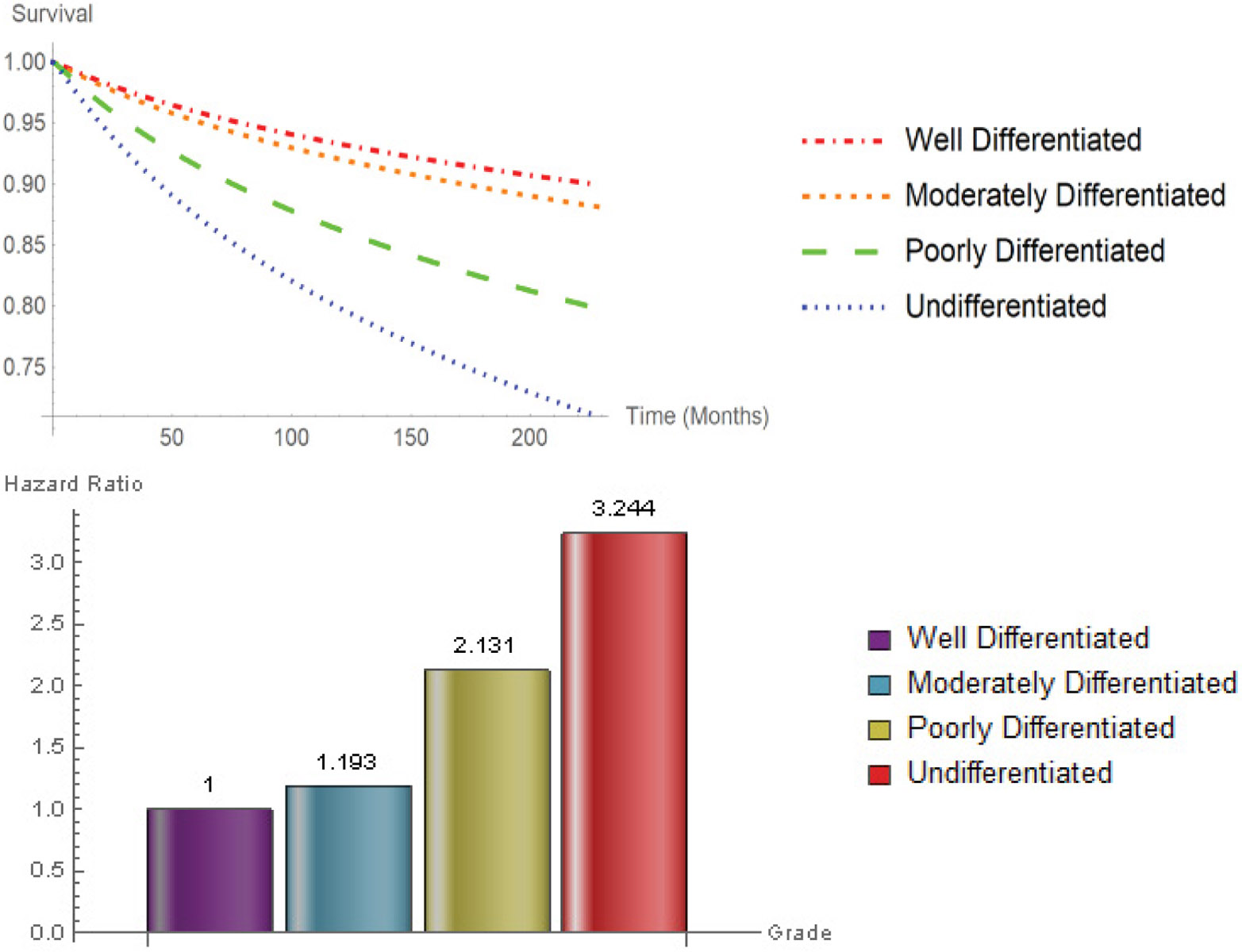
Survival curves hazard ratios for Grade.

Figure 4 displays the survival probabilities and hazard ratios for tumor grade. Disease grade played an important role in determining the patient survival probabilities. The median survival time for well differentiated, moderately differentiated, poorly differentiated, and undifferentiated were 72, 62, 44, and 29 months respectively. Hazard ratios of moderately differentiated, poorly differentiated, and undifferentiated grade are 1.193 (CI: 1.102-1.291), 2.131 (CI: 2.049-2.216), and 3.244 (CI: 3.029-3.475), with respect to well differentiated grade.

The hazard ratio of undifferentiated to well differentiated tumor grade was 3.244.

**Figure 5:**
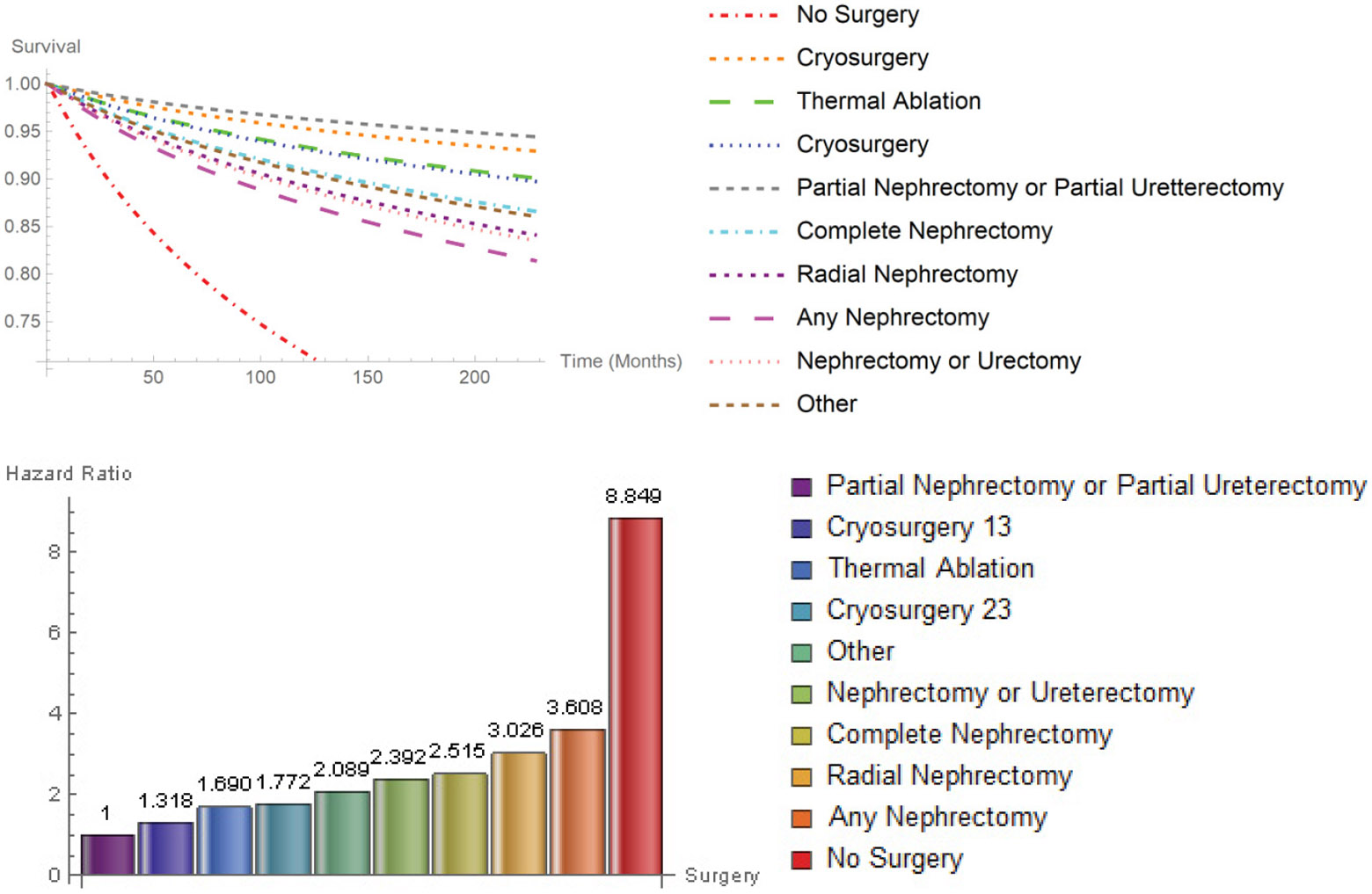
Survival probability hazard ratios for Surgery.

Figure 5 reveals that cryosurgery 13 (HR: 1.318, CI: 0.882-1.971), thermal ablation (HR: 1.690, CI: 1.086-2.631), cryosurgery 23 (HR: 1.772, CI: 1.275-2.463), other types of surgery (HR: 2.089, CI: 1.753-2.489), nephrectomy/ureterectomy (HR: 2.392, CI: 1.949-2.935), complete nephrectomy (HR: 2.515, CI: 2.122-2.981), radical nephrectomy (HR: 3.026, CI: 2.574-3.557), any nephrectomy (HR: 3.608, CI: 3.003-4.335), and no surgery (HR: 8.849, CI: 7.480-10.469) had higher risk of death with respect to patients treated with partial nephrectomy or partial ureterectomy. By far, patients that did not undergo surgery had the worst survival probability. The speed of decline in survival probability for those patients who refused surgery was highest, when compared with those who had any type of surgery at every time point. For every 100 deaths associated with patients who underwent partial nephrectomy or ureterectomy, there will be 885 deaths associated with patients who did not undergo any type of surgery.

**Figure 6:**
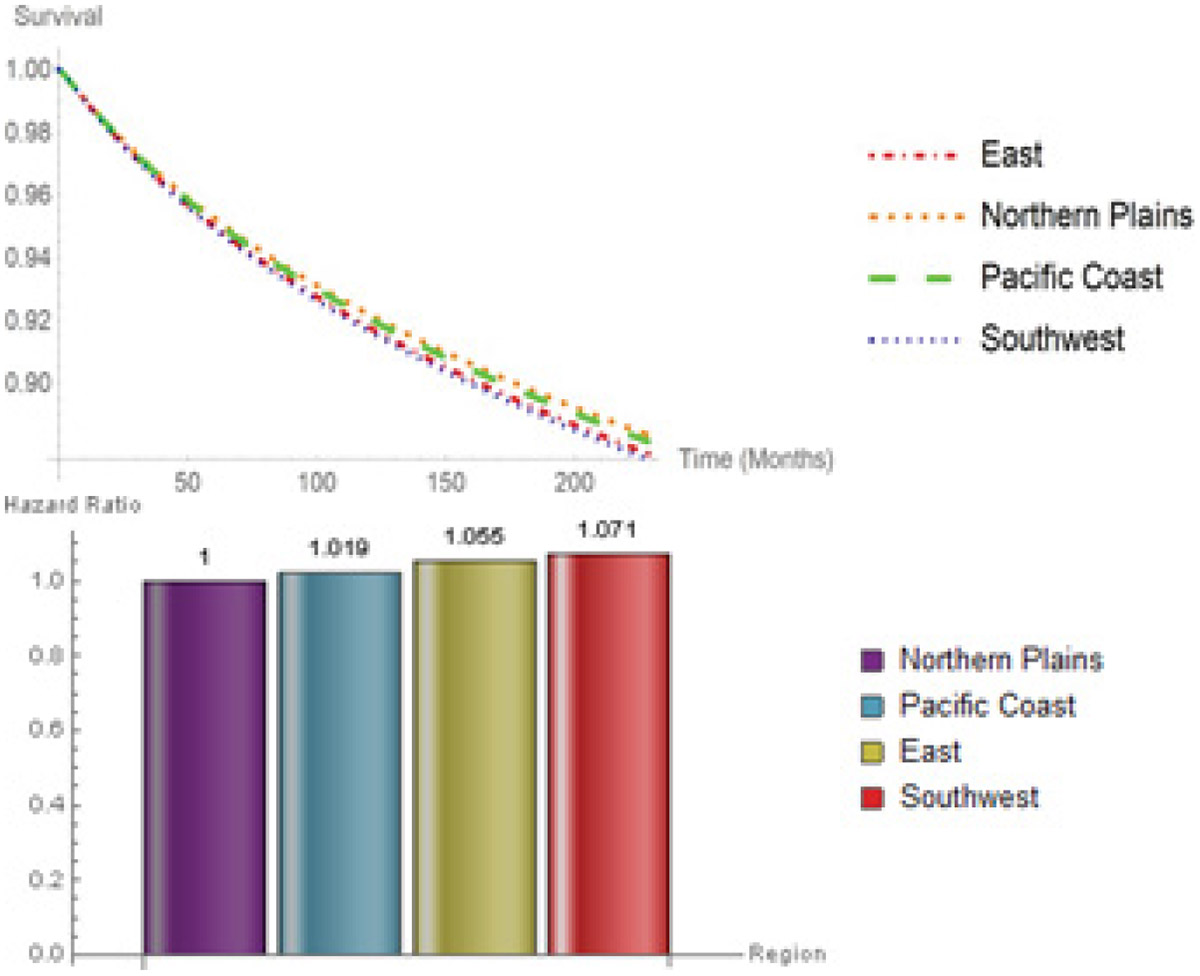
Survival probabilities hazard ratios for Region.

In spite of region being an overall significant factor (*P* < 0.001), a further examination of Figure 6 reveals that there was no significant difference in survival probability in East and Southwest. In addition, there was no significant difference between the survival probability of patients living in Northern Plains and Pacific Coast. Southwest, East, and Pacific Coast hazard ratios are 1.071 (CI: 0.990-1.159), 1.055 (CI: 0.985-1.130), and 1.019 (CI: 0.953-1.090), relative to Northern Plains.

**Figure 7:**
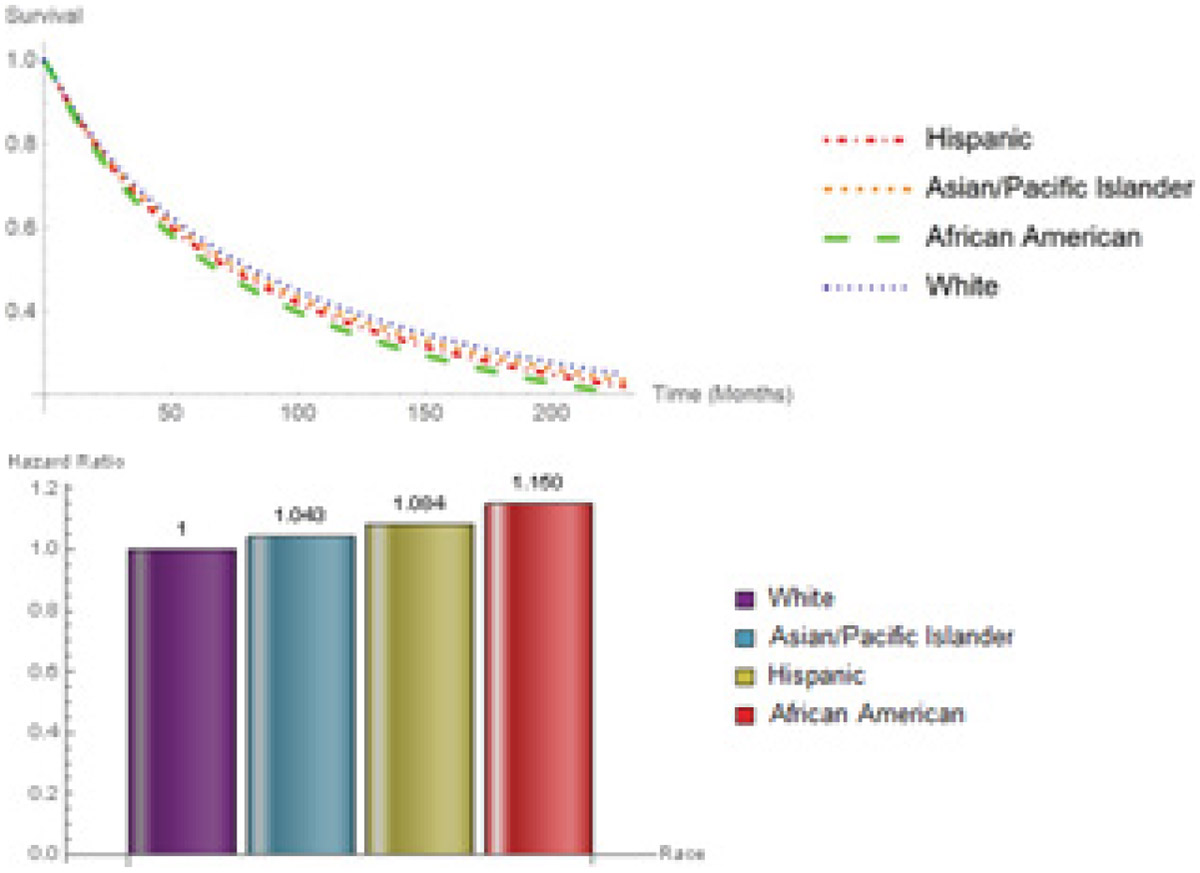
Survival probabilities hazard ratios for Race.

**Figure 8:**
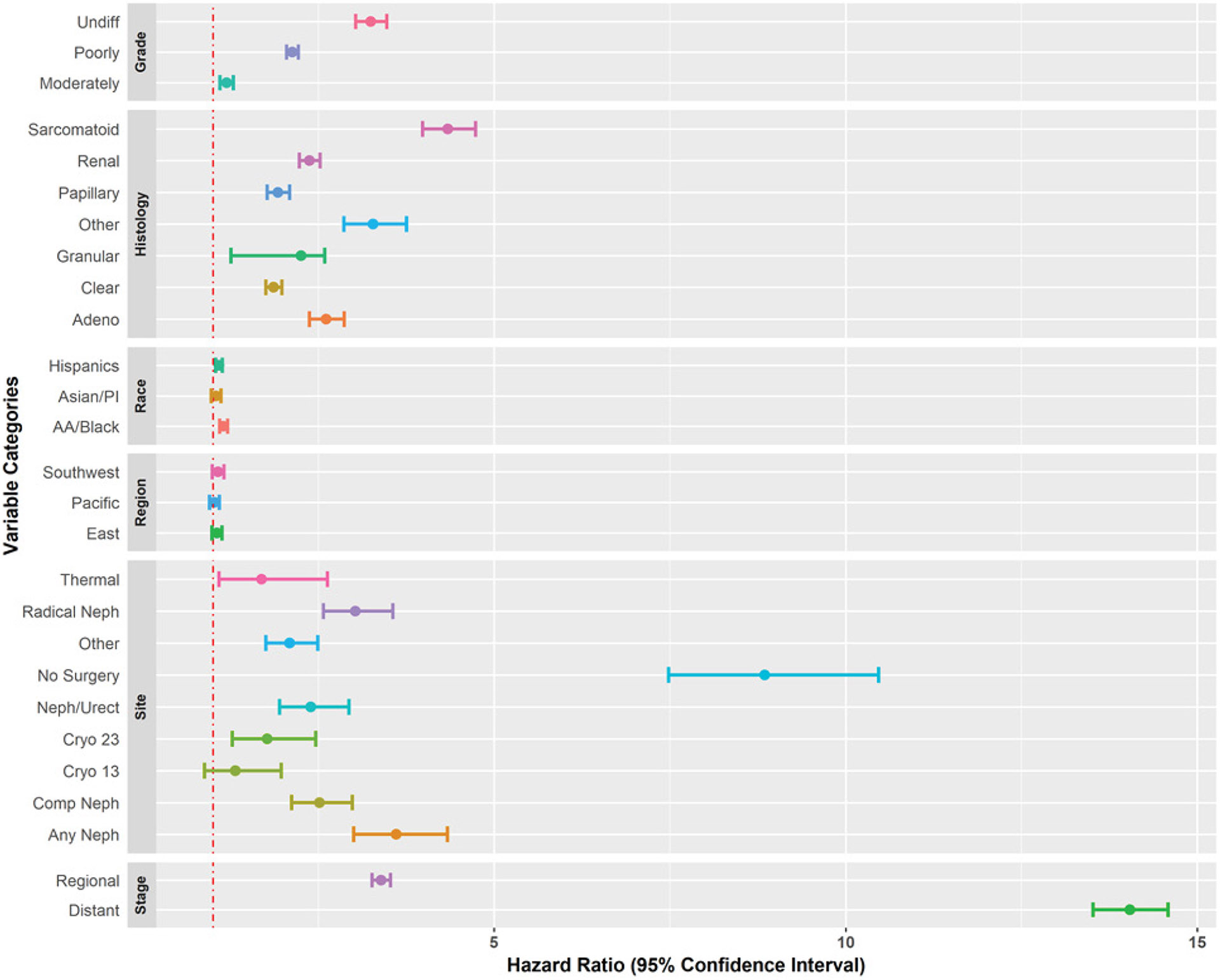
Forest plot of hazard ratios with 95% confidence intervals.

Survival curves and hazard ratios for racial groups are shown in Figure 7. The lowest survival probability belonged to African Americans (HR: 1.15, CI: 1.094-1.208), followed by Hispanics (HR: 1.084, 1.037-1.134), Asian/Pacific Islanders (HR: 1.043, 0.977-1.113), and Whites. There was a significant difference between Hispanics and Whites (P-value < 0.001) as well as African Americans and Whites (P-value < 0.001). Figure 8 illustrates a summary forest plot of hazard ratios and their corresponding 95% confidence intervals for all categorical variables.

**Figure 9:**
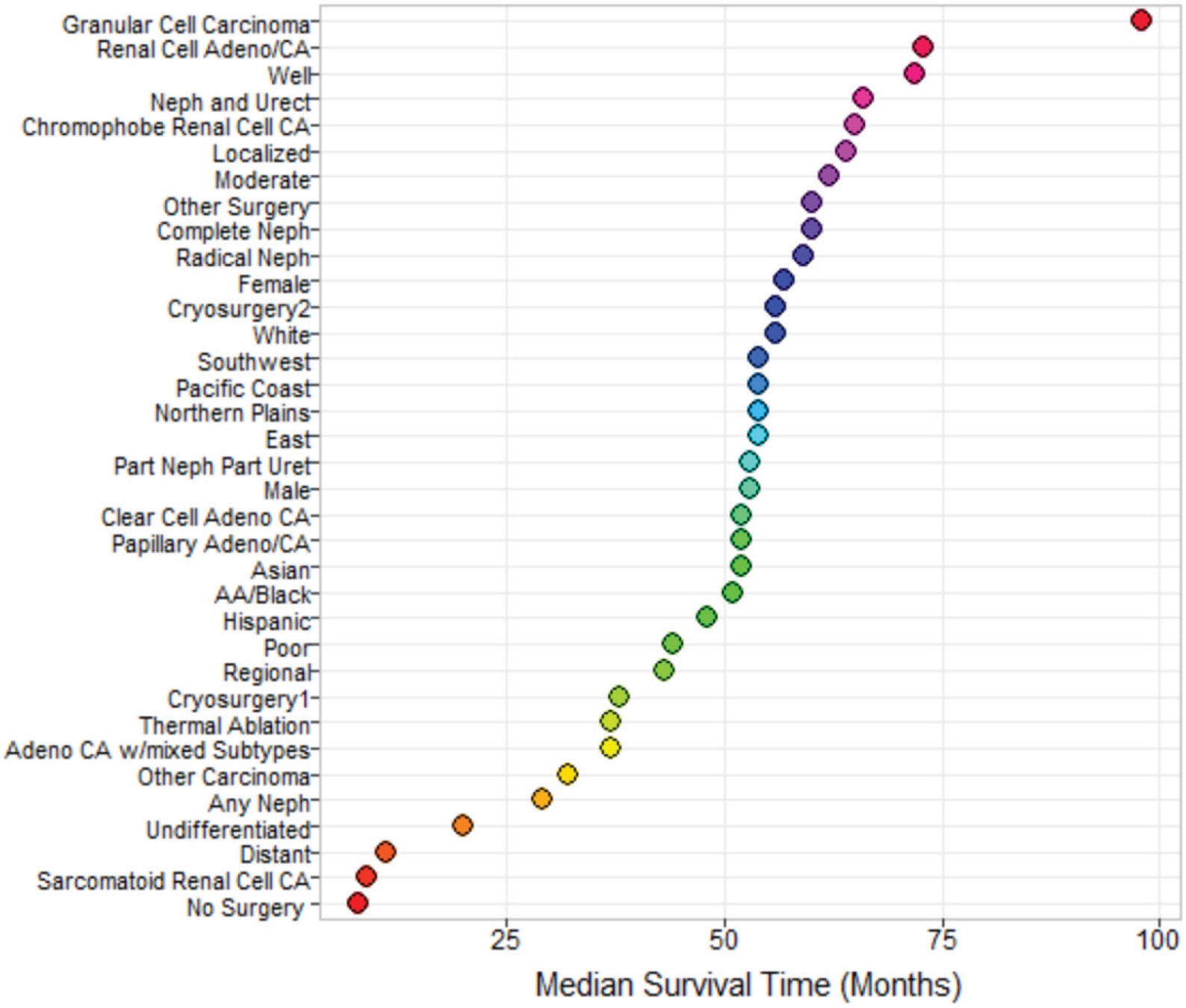
Forest plot for median survival time.

A comparison of median survival times of all categories of model variables, revealed that patients who did not undergo any type of surgery had the lowest median survival time, as indicated by Figure 9. The next lowest median survival time belonged to patients who had sarcomatoid RCC followed closely by individuals who had distant tumor stage. By far, patients who were diagnosed with granular cell carcinoma had the highest median survival time. The 5- and 10- year survival probabilities are depicted for a typical male living in the East region diagnosed with distant stage, undifferentiated grade, and underwent partial nephrectomy or partial ureterectomy surgery, embodied in Table 7.

Male patients diagnosed with sarcomatoid RCC, as indicated in Table 7, had the worst probability of survival among all histological subtypes and all racial groups. The 5- and 10-year survival probabilities for sarcomatoid RCC were [Asian/Pacific Islanders = (0.209, 0.073), Hispanics = (0.196, 0.065), African Americans = (0.178, 0.056), Whites = (0.223, 0.081)]; however, the difference in survival probabilities across racial groups were not significant. African Americans had the lowest survival probability for both 5- and 10- year, when diagnosed with sarcomatoid RCC, and Whites had the highest.

Similarly, Table 8 shows the 5- and 10-year survival probabilities for a typical male living in the East region diagnosed with localized stage, well differentiated grade, and underwent partial nephrectomy or partial ureterectomy surgery; and the survival probabilities were above 93% across all histological subtypes and racial groups. In addition, the survival probabilities for females under the same conditions as males seen in Table 7 and Table 8 had similar results and were excluded from this paper.

Table 9 describes the survival probabilities of male patients living in the East region diagnosed with localized stage, poorly differentiated grade, and underwent radical nephrectomy. Although not shown here, similar results were found for females.

## Discussion

Our analysis of the 134,150 patients who were diagnosed with kidney carcinoma in the United States revealed that histological subtypes play an important role both when used as the sole predictor in the survival model, as well as when controlling for confounding variables. Within all histological subtypes, the majority of patients were diagnosed with clear cell adenocarcinoma (57.8%). Among African American patients, 27.2% were diagnosed with papillary adenocarcinoma NOS, while among Whites, the percentage was 10.1%. In all four regions, the percentage of patients who had clear cell adeno carcinoma were above 50%. Stage was a significant factor with regard to patients’ survival time being 64 months for localized, 43 months for regional, and 11 months for distant. As expected, the survival rate was lowest for patients diagnosed with distant stage [29]. The majority of patients within tumor stage were diagnosed with clear cell adeno carcinoma. Although the percentage of patients diagnosed with clear cell adenocarcinoma for all racial groups was highest when compared with all histological subtypes, the percentages of Whites, Asian/Pacific Islanders, and Hispanics were approximately 1.6, 1.9, and 1.9 times higher than that of African Americans, respectively.

Differences in survival probability, with respect to histological subtypes, can be attributed to several different factors affecting survival. For instance, sarcomatoid RCC is associated with high tumor grade, which can worsen the overall prognosis of patients; chromophobe RCC tends to have a larger cell structure, therefore making it easier to diagnose; and clear cell RCC is infiltrated by a larger number of T cells, resulting in a greater immune response [30-32]. Sarcomatoid RCC patients had a very low survival probability when compared to chromophobe RCC patients. For every 100 deaths due to chromophobe RCC, there will be 434 deaths for sarcomatoid RCC at each time point. Most studies have concentrated on a small number of histological subtypes [22,28,33]. Those afflicted with sarcomatoid RCC had the lowest median survival time of any other category other than those who had no surgery. Data showed patients with this histological subtype tend to be associated with regional and distant stages, suggesting that patients are diagnosed late or are difficult to diagnose [30]. Clear cell adenocarcinoma is by far the leading cancer type in our study, accounting for over half of all patients. The authors suggest more research should be done on these subtypes due to their severity and large frequency.

In the univariate analysis, sex was a significant factor (*P* < 0.001); but in the multivariable model, when controlling for potential confounders, the effect of sex disappeared (*P* < 0.306). Similar results were previously observed [34]. Our results indicated that Whites had the highest probability of surviving death due to kidney carcinoma, followed by Asian/Pacific Islanders and Hispanics. African Americans had the lowest overall survival probabilities. Among histological subtypes, chromophobe renal cell adeno carcinoma had the highest survival probability followed by clear cell adenocarcinoma. The lowest survival probability was associated with sarcomatoid RCC.

This study obtained appropriate variables from a database consistently maintaining current and reliable big data. Furthermore, the results will help to further research in needed areas. The Hypertabastic survival model used here has flexibility in shaping hazard curves when compared to classical models [3,6]. Identification of histological subtypes are important in prognosis and precision medicine [35]. One of the limitations in this study is that patients in the SEER database tend to be urban and belong to a lower socioeconomic status [36]. To assess the risk associated with different combinations of factors, we have provided a R program in the supplementary material section. The authors believe that this paper will assist health care providers in making wise decisions in the future treatment of kidney carcinoma patients by examining the risk associated with different combinations of age, race/ethnicity, sex, tumor grade, type of surgery, geographical location of patient, and stage of disease.

## Supplementary Material

Kidney Carcinoma R Program

## Figures and Tables

**Table 1:** Frequencies and median survival times for patients with indicated histological subtypes.

ICD-O-3 Code	Text Description	Freq	Median Survival (Month)	ICD-O-3 Code	Text Description	Freq	Median Survival (Month)
**8310**	Clear Cell Adeno/Ca	77547	52	**8020**	Undifferentiated Carcinoma	11	2
**8312**	Renal Cell Adeno/Ca	26368	73	**8021**	Anaplastic Carcinoma	6	4.5
**8260**	Papillary Adeno Ca NOS	14860	52	**8481**	Mucin Prod Ca/Adenoc	6	17
**8317**	Chromophobe Renal Cell Ca	5174	65	**8280**	Acidophil Ca/Adenoc	5	107
**8255**	Adeno CA w/mixed subtypes	3332	37	**8313**	Clear cell Adeno carcino fibroma	5	20
**8318**	Sarcomatoid Renal Cell Ca	1505	9	**8013**	Lg Cell Neuroendocrine Carc	4	9.5
**8320**	Granular Cell Carcinoma	990	98	**8342**	Pap Carcinoma Oxyphilic cell	4	39
**8120**	Transitional Cell Carcinoma	936	12	**8074**	Squam Cell Ca Spindle Cell	3	3
**8316**	Cyst-assoc renal cell Ca	796	81	**8230**	Solid Carcinoma NOS	3	94
**8130**	Papillary Transitional Cell Ca	691	47	**8504**	Intracyst Pap Aden/Ca	3	149
**8319**	Collecting Duct Carcinoma	314	17	**8560**	Adeno squamous Carcinoma	3	5
**8050**	Papillary Squamous Cell Carc	286	78	**8022**	Pleomorphic Carcinoma	2	73.5
**8010**	Carcinoma NOS	237	5	**8031**	Giant Cell Carcinoma	2	51
**8140**	Adeno Carcinoma NOS	225	19	**8052**	Papillary Carcinoma	2	34
**8323**	Mixed Cell Carcinoma	133	52	**8072**	Squamous Cell Ca non-Kerit	2	5.5
**8270**	Chromophobe Adeno/Ca	101	72	**8131**	Micropapillary Transitnl Cell Ca	2	34
**8290**	Oncocytic Adeno/Ca	96	68.5	**8190**	Trabecular Adeno/Ca	2	123
**8070**	Squamous Cell Carcinoma	81	6	**8330**	Follicular Adeno/Ca	2	53
**8033**	Pseudosarcomatous Carcinoma	55	5	**8522**	Mix Duct & Lobular Ca	2	9
**8510**	Medullary Adeno/Ca	47	7	**8574**	Aden/Ca; Neuroendocrine diff	2	5.5
**8211**	Tubular Adeno/Ca	44	93	**8030**	Giant & Spindle Cell Carc	1	NA
**8480**	Mucinous Ca/Adenoc	44	26.5	**8076**	Squam Cell Ca: Microinvasive	1	NA
**8246**	Neuroendocrine Ca	33	22	**8083**	Basaloid Squamous Cell Carc	1	NA
**8041**	Small Cell Carcinoma NOS	28	6	**8210**	Adenoca in Aden Polyp	1	NA
**8032**	Spindle Cell Carc	22	10	**8249**	Atypical Carcinoid Tumor	1	NA
**8240**	Carcinoid Tumor	21	67	**8251**	Alveolar Adeno/Ca	1	NA
**8263**	Adenoca in Tubulovill Ad	21	101	**8315**	Glycogen-Rich Carc	1	NA
**8046**	Non-Small Cell Carc	19	2	**8325**	Metanephric adenoma	1	NA
**8071**	Squamous Cell Ca Keratiniz	17	9	**8440**	Cystadenocarcinoma NOS	1	NA
**8550**	Acinic Cell Adeno/Ca	17	99	**8490**	Signet Ring Cell Adeno/Ca	1	NA
**8122**	Spindle Cell Transitional Cell Ca	15	6	**8500**	Duct Adeno/Ca	1	NA
**8012**	Large Cell Carcinoma NOS	12	7	**8503**	Intraduc Pap Adeno/Ca	1	NA

**Table 2:** Kidney Carcinoma Histological Subtypes by Tumor Stage.

	Localized		Regional		Distant	
	% Within Hist	% Within Tumor	% Within Hist	% Within Tumor	% Within Hist	% Within Tumor
**Adeno carcinoma with mixed type**	66.1	2.2	18.8	2.7	15.1	4.3
**Papillary adeno carcinoma NOS**	85.6	12.8	11.2	7.1	3.3	4.2
**Clear cell adeno carcinoma**	74.6	58.2	17.8	59.3	7.7	51.2
**Renal cell adeno carcinoma**	71.7	19.0	17.4	19.8	10.9	24.6
**Chromophobe RCC**	83.9	4.4	13.9	3.1	2.1	0.9
**Sarcomatoid RCC**	18.1	0.3	34.8	2.3	47.2	6.1
**Granular cell carcinoma**	66	0.7	23.90	1.0	10.10	0.9
**Other histological subtypes**	54.1	2.4	25.2	4.8	20.7	7.8

* Percentages may not add up to 100% due to rounding.

**Table 3:** Kidney carcinoma histological subtypes by race.

	African Americans		Whites		Asian/Pacific Islanders		Hispanics	
	% Within Hist	% Within Race	% Within Hist	% Within Race	% Within Hist	% Within Race	% Within Hist	% Within Race
**Adenocarcinoma with mixed type**	20.9	4.6	63.9	2.3	4.5	2.2	10.7	2.0
**Papillary adenocarcinoma NOS**	27.6	27.2	64.1	10.1	2.8	6.1	5.6	4.6
**Clear cell adenocarcinoma**	7.0	36.1	71.4	58.6	5.9	68.3	15.6	67.9
**Renal cell adenocarcinoma**	12.2	21.4	71.9	20.1	3.8	14.9	12.0	17.8
**Chromophobe RCC**	13.8	4.7	69.5	3.8	4.7	3.6	12.0	3.5
**Sarcomatoid RCC**	9.5	0.9	70.6	1.1	6.1	1.4	13.8	1.2
**Granular cell carcinoma**	10.8	0.7	73.4	0.8	4.2	0.6	11.5	0.6
**Other histological subtypes**	14.6	4.2	71.2	3.3	4.4	2.9	9.8	2.4
**Adenocarcinoma with mixed type**	20.9	4.6	63.9	2.3	4.5	2.2	10.7	2.0
**Papillary adenocarcinoma NOS**	27.6	27.2	64.1	10.1	2.8	6.1	5.6	4.6

* Percentages may not add up to 100% due to rounding

**Table 4:** Kidney carcinoma histological subtypes by region.

	East		Northern Plains		Pacific Coast		Southwest	
	% Within Hist	% Within Region	% Within Hist	% Within Region	% Within Hist	% Within Region	% Within Hist	% Within Region
**Adenocarcinoma with mixed type**	36.4	2.3	12.3	2.8	49.1	2.7	2.2	1.2
**Papillary adenocarcinoma NOS**	44.8	12.6	13.1	13.5	39.2	9.6	2.9	7.2
**Clear cell adenocarcinoma**	34.1	50.0	12.1	65.1	49.1	62.5	4.7	61.2
**Renal cell adenocarcinoma**	52.2	26.1	5.9	10.8	37.2	16.1	4.7	20.8
**Chromophobe RCC**	38.0	3.7	9.4	3.4	47.6	4.0	5.0	4.3
**Sarcomatoid RCC**	38.7	1.1	10.3	1.1	46.5	1.1	4.5	1.1
**Granular cell carcinoma**	27.3	0.5	10.4	0.7	57.0	0.9	5.4	0.9
**Other histological subtypes**	43.9	3.6	8.4	2.5	43.3	3.1	4.4	3.2
**Adeno carcinoma with mixed type**	36.4	2.3	12.3	2.8	49.1	2.7	2.2	1.2
**Papillary adeno carcinoma NOS**	44.8	12.6	13.1	13.5	39.2	9.6	2.9	7.2

* Percentages may not add up to 100% due to rounding.

**Table 5:** Kidney carcinoma histological subtypes by grade.

	Well Differentiated		Moderately Differentiated		Poorly Differentiated		Undifferentiated	
	% Within Hist	% Within Grade	% Within Hist	% Within Grade	% Within Hist	% Within Grade	% Within Hist	% Within Grade
**Adenocarcinoma with mixed type**	10.8	2.0	40.2	2.0	29.3	2.6	19.7	6.4
**Papillary adenocarcinoma NOS**	12.8	10.5	53.1	11.6	30.9	12.1	3.3	4.8
**Clear cell adenocarcinoma**	13.4	57.3	53.6	61.2	26.7	54.7	6.2	47.3
**Renal cell adenocarcinoma**	16.2	23.5	47.7	18.5	28.4	19.8	7.8	20.0%
**Chromophobe RCC**	8.4	2.4	54.3	4.1	31.6	4.3	5.6	2.9
**Sarcomatoid RCC**	1.7	0.1	4.9	0.1	27.3	1.1	66.1	9.7
**Granular cell carcinoma**	7.9	0.4	40.8	0.6	39.1	1.0	12.2	1.2
**Other histological subtypes**	15.5	3.7	28.5	1.8	38.0	4.4	18.0	7.7
**Adeno carcinoma with mixed type**	10.8	2.0	40.2	2.0	29.3	2.6	19.7	6.4
**Papillary adeno carcinoma NOS**	12.8	10.5	53.1	11.6	30.9	12.1	3.3	4.8

* Percentages may not add up to 100% due to rounding

**Table 6:** Kidney Carcinoma Surgical Type by Histological Subtypes.

	Adeno carcinoma with mixed type		Papillary adeno CA NOS		Clear cell adeno CA		Renal cell adeno CA		Chromophobe		Sarcomatoid RCC		Granular cell CA		Other histological types	
	% Within Hist	% Within Surgery	% Within Hist	% Within Surgery	% Within Hist	% Within Surgery	% Within Hist	% Within Surgery	% Within Hist	% Within Surgery	% Within Hist	% Within Surgery	% Within Hist	% Within Surgery	% Within Hist	% Within Surgery
**No Surgery**	2.2	1.4	2.4	6.6	3.0	43.3	6.4	31.6	1.1	1.0	10.9	3.1	3.2	0.6	15.0	12.4
**Cryosurgery 13**	0.7	1.9	1.2	14.3	1.1	64.3	0.8	16.6	0.4	1.7	0.1	0.1	0.3	0.2	0.3	0.9
**Thermal ablation**	0.3	1.2	0.8	15.6	0.7	67.0	0.4	13.4	0.3	2.1	0.0	0.0	0.0	0.0	0.1	0.8
**Cryosurgery 23**	0.7	1.7	1.1	12.7	1.1	64.0	0.9	17.7	0.5	2.0	0.0	0.0	1.1	0.8	0.3	1.1
**Partial Nephrectomy/Ureterectomy**	26.1	2.4	41.4	16.7	28.5	59.9	19.6	14.0	33.1	4.6	5.4	0.2	13.3	0.4	15.7	1.9
**Complete Nephrectomy**	7.1	2.3	7.9	11.1	7.5	55.0	7.5	18.7	7.6	3.8	5.3	0.8	6.6	0.6	18.7	7.8
**Radical Nephrectomy**	59.5	2.7	43.0	8.6	56.3	58.5	60.9	21.5	55.0	3.8	70.8	1.4	72.8	1.0	44.3	2.6
**Any Nephrectomy**	1.9	4.8	0.7	8.1	0.7	44.1	1.2	24.8	0.9	3.3	6.0	6.8	0.8	0.6	2.2	7.4
**Nephrectomy, Ureterectomy**	1.0	2.9	0.7	8.6	0.7	43.0	1.5	32.3	0.7	3.2	1.1	1.3	1.4	1.2	2.1	7.6
**Other**	0.5	1.9	0.8	13.0	0.5	46.4	1.0	28.7	0.4	2.3	0.5	0.9	0.4	0.5	1.3	6.3

* Percentages may not add up to 100% due to rounding.

**Table 7:** Survival probabilities for a typical male living in the East region diagnosed with distant stage, undifferentiated grade, and underwent partial nephrectomy or partial ureterectomy surgery.

Race/Ethnicity								
	Asian/PI		Hispanics		African American		Whites	
Histological Subtype	5-YRS	10-YRS	5-YRS	10-YRS	5-YRS	10-YRS	5-YRS	10-YRS
Adenocarcinoma With mixed subtypes	0.390	0.207	0.376	0.194	0.354	0.176	0.406	0.221
Papillary Adenocarcinoma NOS	0.500	0.313	0.486	0.299	0.465	0.278	0.514	0.328
Clear Cell Adenocarcinoma	0.511	0.325	0.497	0.310	0.477	0.289	0.525	0.340
Renal Cell Adenocarcinoma	0.425	0.239	0.411	0.226	0.390	0.206	0.440	0.253
Chromophobe Renal Cell Carcinoma	0.697	0.547	0.687	0.534	0.672	0.514	0.708	0.560
Sarcomatoid Renal Cell Carcinoma	0.209	0.073	0.196	0.065	0.178	0.056	0.223	0.081
Granula Cell Carcinoma	0.444	0.257	0.430	0.243	0.409	0.224	0.459	0.272
Other Histological Subtypes	0.307	0.138	0.293	0.128	0.272	0.113	0.322	0.150

**Table 8:** Survival probabilities for a typical male living in the East region diagnosed with localized stage, well differentiated grade, and underwent partial nephrectomy or partial ureterectomy surgery.

Race/Ethnicity								
	Asian/PI		Hispanics		African American		Whites	
Histological Subtype	5-YRS	10-YRS	5-YRS	10-YRS	5-YRS	10-YRS	5-YRS	10-YRS
Adenocarcinoma With mixed subtypes	0.980	0.966	0.979	0.965	0.977	0.963	0.980	0.967
Papillary Adenocarcinoma NOS	0.985	0.975	0.984	0.974	0.983	0.972	0.985	0.976
Clear Cell Adenocarcinoma	0.985	0.976	0.985	0.975	0.984	0.973	0.986	0.977
Renal Cell Adenocarcinoma	0.981	0.970	0.981	0.968	0.980	0.966	0.982	0.970
Chromophobe Renal Cell Carcinoma	0.992	0.987	0.992	0.986	0.991	0.985	0.992	0.987
Sarcomatoid Renal Cell Carcinoma	0.966	0.944	0.965	0.942	0.963	0.939	0.968	0.946
Granula Cell Carcinoma	0.982	0.971	0.982	0.969	0.981	0.968	0.983	0.972
Other Histological Subtypes	0.974	0.957	0.973	0.956	0.972	0.953	0.975	0.959

**Table 9:** Survival probabilities for a typical male living in the East region diagnosed with localized stage, poorly differentiated grade, and underwent radical nephrectomy surgery.

Race/Ethnicity								
	Asian/PI		Hispanics		African American		Whites	
Histological Subtype	5-YRS	10-YRS	5-YRS	10-YRS	5-YRS	10-YRS	5-YRS	10-YRS
Adenocarcinoma With mixed subtypes	0.875	0.800	0.871	0.793	0.863	0.782	0.880	0.807
Papillary Adenocarcinoma NOS	0.906	0.848	0.903	0.843	0.897	0.834	0.910	0.854
Clear Cell Adenocarcinoma	0.909	0.853	0.906	0.847	0.900	0.839	0.913	0.858
Renal Cell Adenocarcinoma	0.886	0.816	0.882	0.810	0.875	0.800	0.890	0.823
Chromophobe Renal Cell Carcinoma	0.950	0.918	0.948	0.915	0.945	0.910	0.952	0.921
Sarcomatoid Renal Cell Carcinoma	0.801	0.690	0.794	0.680	0.783	0.664	0.808	0.700
Granula Cell Carcinoma	0.891	0.825	0.887	0.819	0.881	0.809	0.896	0.831
Other Histological Subtypes	0.846	0.756	0.840	0.747	0.831	0.734	0.852	0.764
